# Laboratory markers associated with COVID‐19 progression in patients with or without comorbidity: A retrospective study

**DOI:** 10.1002/jcla.23644

**Published:** 2020-10-28

**Authors:** Zaishu Chen, Furong Zhang, Weihua Hu, Qijian Chen, Chang Li, Longlong Wu, Zhuheng Zhang, Bin Li, Qifa Ye, Jin Mei, Jiang Yue

**Affiliations:** ^1^ Jiayu People's Hospital Jiayu China; ^2^ Department of Pharmacology Wuhan University School of Basic Medical Sciences Wuhan China; ^3^ Jingzhou First People's Hospital Jingzhou China; ^4^ Emergency Department Fifth Hospital in Wuhan Wuhan China; ^5^ Hubei No.3 People's Hospital of Jianghan University Wuhan China; ^6^ People's Hospital of Nanzhang County Xiangyang China; ^7^ Zhongnan Hospital of Wuhan University Institute of Hepatobiliary Diseases of Wuhan University Wuhan China; ^8^ Central Laboratory Ningbo First Hospital Zhejiang University Ningbo China; ^9^ Hubei Province Key Laboratory of Allergy and Immunology Wuhan China

**Keywords:** Coronavirus disease‐19 (COVID‐19), diabetes, hypertension, lactate dehydrogenase, liver diseases

## Abstract

**Objectives:**

To investigate laboratory markers for COVID‐19 progression in patients with different medical conditions.

**Methods:**

We performed a multicenter retrospective study of 836 cases in Hubei. To avoid the collinearity among the indicators, principal component analysis (PCA) followed by partial least squares discriminant analysis (PLS‐DA) was performed to obtain an overview of laboratory assessments. Multivariable logistic regression analysis and multivariable Cox proportional hazards regression analysis were respectively used to explore risk factors associated with disease severity and mortality. Survival analysis was performed in patients with the most common comorbidities.

**Results:**

Lactate dehydrogenase (LDH) and prealbumin were associated with disease severity in patients with or without comorbidities, indicated by both PCA/PLS‐DA and multivariable logistic regression analysis. The mortality risk was associated with age, LDH, C‐reactive protein (CRP), D‐dimer, and lymphopenia in patients with comorbidities. CRP was a risk factor associated with short‐term mortality in patients with hypertension, but not liver diseases; additionally, D‐dimer was a risk factor for death in patients with liver diseases.

**Conclusions:**

Lactate dehydrogenase was a reliable predictor associated with COVID‐19 severity and mortality in patients with different medical conditions. Laboratory biomarkers for mortality risk were not identical in patients with comorbidities, suggesting multiple pathophysiological mechanisms following COVID‐19 infection.

## Graphical abstract

Lactate dehydrogenase was a reliable predictor associated with COVID‐19 severity and mortality. Mortality risk factors were not identical in COVID‐19 patients with comorbidities. CRP was related with death risk in patients with hypertension, but not liver diseases. D‐dimer was an independent risk factor for death in patients with liver diseases.

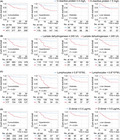

**Abbreviations**
APTT: activated partial thromboplastin time
CK: creatine kinase
COVID‐19: Coronavirus disease‐19
CRP: c‐reactive protein
CT: chest computed tomographic
DBIL: direct bilirubin
GGT: γ‐glutamyl transpeptidase
LDH: lactate dehydrogenase
LSD: least significant difference
PCA: principal component analysis
PLS‐DA: partial least squares discriminant analysis
PLT: platelets
TBIL: total bilirubin

## 1 INTRODUCTION

The COVID‐19 pandemic has spread rapidly across the world. By July 13, more than 13 million people were infected with COVID‐19 across the world, and over half a million people have died, according to the data provided by Johns Hopkins University. The risk classification based on the laboratory markers can be helpful to provide early intervention and reduce mortality of COVID‐19 patients. However, the reported laboratory biomarkers associated with disease progression were inconsistent.^1^, ^2^, ^3^, ^4^ The type and distribution of comorbidities in the studied cohorts varied,^5^ which may affect the biomarker analysis as the comorbidities are the leading cause for the conversion to severe and critical cases in COVID‐19 patients. The features of laboratory indicators in specific subgroups remain ambiguous, although clinical characteristics of COVID‐19 patients have been documented.^6^, ^7^, ^8^

We performed a retrospective and multisite study of COVID‐19 cases from designated tertiary hospitals in Hubei province to investigate the association between laboratory indicators and disease progression (severity and mortality) in patients with different medical conditions. In order to avoid the collinearity among the indicators, principal component analysis (PCA) followed by partial least squares discriminant analysis (PLS‐DA) was respectively performed in patients with or without comorbidity. Multivariate logistic regression model was respectively used to explore the risk factors associated with disease severity using the variables extracted from the PCA/PLS‐DA analysis. The association between laboratory makers at admission and short‐term survival rate was analyzed in patients with the most common comorbidities including hypertension, diabetes, and liver diseases. The data may yield a novel insight for the progression predictors of COVID‐19, informing the therapy management and the pathophysiological mechanism involved in COVID‐19 infection.

## 2 METHODS

### 2.1 Study design

This was a retrospective study that included patients (age 18‐90) from the Jiayu People's Hospital, Jingzhou First People's Hospital, Fifth Hospital in Wuhan, Hubei No.3 People's Hospital of Jianghan University, People's Hospital of Nanzhang County, and People's Hospital of Nanzhang County in Hubei province from January 16 2020 to March 21 2020. All the cases were diagnosed by a real‐time RT‐PCR assay using nasal and pharyngeal swab specimens, the confirmatory testing for COVID‐19 according to the WHO interim guidance.^9^

A total of 836 cases with intact laboratory information were included in the study, and 615 patients were categorized as mild/moderate cases, while 221 individuals were categorized as severe/critical cases. Of 615 mild/moderate cases, 158 patients were identified with no comorbidities. Of 221 severe/critical cases, 43 patients were identified with no comorbidities. No statistical method was used to predetermine the sample size. The study was approved by the institutional board of People's Hospital of Jiayu County. The written informed consent was waived because of the urgent.

### 2.2 Data collection

We collected the information including demographic, epidemiological, clinical, laboratory, treatment, and outcome data. A standardized case report form was used to extract medical records. The epidemiological characteristics (including recent exposure history), clinical symptoms and signs, and laboratory findings at the time point for the first hospital admission were extracted from electronic medical records. Laboratory assessments consisted of complete blood count, blood chemistry, coagulation test, liver and renal function, electrolytes, C‐reactive protein (CRP), lactate dehydrogenase (LDH), creatine kinase, and chest computed tomographic (CT).

### 2.3 Study definition and primary outcome

The categories of COVID‐19 cases were defined according to the diagnosis and treatment protocol for novel coronavirus pneumonia (Trial Version 5) by National Health Commission & State Administration of Traditional Chinese Medicine.^10^ Mild case refers to the clinical symptoms were mild, and there was no sign of pneumonia on imaging. Moderate case refers to showing fever and respiratory symptoms with radiological findings of pneumonia. Severe case meets any of the following criteria: (a) respiratory distress (≥30 breaths/min); (b) oxygen saturation ≤ 93% at rest; (c) arterial partial pressure of oxygen to fraction of inspired oxygen ratio < 300. Critical case meets any of the following criteria: (a) respiratory failure and requiring mechanical ventilation; (b) shock; (c) with other organ failure that requires ICU care.

To assess risk factors associated with disease severity of COVID‐19, the cases were classified according to the diagnostic criteria (mild/moderate cases versus severe/critical cases). For the evaluation of risk factors associated with disease mortality, patients were followed up for a maximum of 30 days after the diagnosis of COVID‐19. Survival time was defined as the time from hospitalization until the occurrence of death.

### 2.4 Statistical analysis

Continuous variables were expressed as medians and interquartile ranges (IQR). Categorical variables were summarized as counts and percentages. One‐way ANOVA followed by the least significant difference (LSD) post hoc test was used to test the differences among the groups for continuous variables when the data were normally distributed; otherwise, the Mann‐Whitney test was used. For categorical variables, χ2 test was used to test the differences among the groups. Principal component analysis (PCA) followed by partial least squares discriminant analysis (PLS‐DA) was performed using SIMCA package (Ver 13.0) (Umetrics, Umea, Sweden) to obtain an overview of laboratory assessments. The principal components were analyzed to identify the important indicators which accounted for the majority of the variation among the groups. PCA was used to analyze the major sources of variation in a multi‐dimensional dataset without introducing inherent bias. PLS‐DA is helpful to class information to maximize the separation between the groups. To explore the risk factors associated with disease severity, we performed multivariable logistic regression analysis by using SPSS 26.0 for Windows (SPSS, Inc, Chicago, Ill). Multivariable Cox proportional hazards regression analysis was to estimate the effects of risk factors on disease mortality. The odds ratio (OR) and hazard ratio (HR) along with the 95% confidence interval (CI) were respectively reported. Survival in patients with comorbidities was estimated by the Kaplan‐Meier method, and any differences in 30‐day survival rate were evaluated with a stratified log‐rank test. A two‐sided α value less than 0.05 was considered statistically significant.

## 3 RESULTS

Laboratory markers at admission associated with disease severity in COVID‐19 patients with no comorbidities. The median age in patients without comorbidities was 46 years (IQR, 36 to 57), and 75.1% of the patients had fever at the time point of first admission to hospital (Table 1). A total of 21.4% patients were categorized as severe/critical cases. The radiologic findings at admission revealed that the most common pattern on chest CT was bilateral patchy shadowing in the adult inpatients (63.2%). Supplementary materials showed the treatment and outcome (Table S1). Among the adult cases, 57.7% of inpatients received oxygen therapy, and mechanical ventilation was administered in 3%.

**Table 1:** Clinical characteristics for adult inpatients without comorbidities. Values are medians (interquartile ranges) or numbers (%)

	Adults			
	Total (n = 201)	Mild and moderate (n = 158)	Severe and critical (n = 43)	*P* value
Epidemiological data				
Age, y	46 (36‐57)	44 (35‐55)	55 (39‐62)	.003
Female	107 (53.2%)	87 (55.1%)	20 (46.5%)	.319
Source of infection				
Family source	23 (11.4%)	20 (12.7%)	3 (7%)	.299
Community source	3 (1.5%)	2 (1.3%)	1 (2.3%)	.611
Nosocomial infection	3 (1.5%)	2 (1.3%)	1 (2.3%)	.611
Others	172 (85.6%)	134 (84.8%)	38 (88.4%)	.556
Hospitalization time, d	18 (11‐25)	17 (10‐22)	21 (12.25‐31)	.016
Onset time, d	31 (22‐37)	30 (21.5‐37)	33 (22.5‐39)	.479
Prodromal symptoms and signs				
Fever	151 (75.1%)	111 (70.3%)	40 (93%)	.002
Dry cough	103 (51.2%)	77 (48.7%)	26 (60.5%)	.172
Expectoration	28 (13.9%)	20 (12.7%)	8 (18.6%)	.318
Shortness of breath	35 (17.4%)	23 (14.6%)	12 (27.9%)	.041
Diarrhea	20 (10%)	17 (10.8%)	3 (7%)	.463
Laboratory tests				
Abnormalities on chest CT				
Ground‐glass opacity	45 (22.4%)	30 (19%)	15 (34.9%)	.027
Local patchy shadowing	23 (11.4%)	22 (13.9%)	1 (2.3%)	.034
Bilateral patchy shadowing	127 (63.2%)	99 (62.7%)	28 (65.1%)	.767
Interstitial abnormalities	5 (2.5%)	5 (3.2%)	0	.237
White blood cell count, 10^9^/L	5.3 (4.0‐6.8)	5.3 (4.2‐6.8)	5.1 (3.6‐7.8)	.469
Neutrophils, 10^9^/L	3.4 (2.4‐4.6)	3.3 (2.3‐4.5)	3.8 (2.4‐6)	.059
Lymphocytes, 10^9^/L	1.3 (1‐1.8)	1.4 (1.1‐2)	0.9 (0.7‐1.3)	<.001
<1 (n)	55	30	25	<.001
Eosinophils, 10^9^/L	0.04 (0.01‐0.09)	0.05 (0.01‐0.09)	0.01 (0‐0.05)	.018
Basophils, 10^9^/L	0.02 (0.01‐0.03)	0.02 (0.01‐0.03)	0.01 (0.01‐0.03)	.62
Red blood cells, 10^12^/L	4.4 (4.1‐4.8)	4.4 (4.1‐4.7)	4.3 (3.8‐4.9)	.614
Hemoglobin, g/L	131.5 (122‐143)	132 (123‐143)	130 (115‐143)	.471
Platelets, 10^9^/L	202 (152‐268.5)	203 (160.30‐268.3)	180 (139‐280)	.457
C‐reactive protein, mg/L	6.1 (1‐24.5)	2.7 (1‐19.9)	16.1 (8.7‐50.5)	.008
>5 (n)	107	69	38	<.001
Prothrombin time, s	12.6 (11.1‐14.3)	12.5 (11.‐14.2)	13 (11.7‐15.4)	.008
International normalized ratio	1 (0.9‐1.1)	1.0 (0.9‐1.1)	1.1 (1.0‐1.2)	<.001
Activated partial thromboplastin time, s	34.4 (27‐39.4)	34.1 (26.8‐38.6)	36.2 (30.0‐42.7)	.018
Fibrinogen, g/L	3.5 (2.6‐4.5)	3.1 (2.5‐4.3)	4.3 (3.7‐5.2)	<.001
>4 (n)	74	49	25	.001
D‐dimer, μg/mL	0.4 (0.2‐0.8)	0.4 (0.2‐0.6)	1.1 (0.4‐2.7)	.015
>0.5 (n)	8	2	6	<.001
Thrombin time, s	17.3 (16.6‐18.1)	17.3 (16.6‐18.1)	17.7 (16.6‐18.4)	.697
Fibrinogen degradation products, μg/mL	2.8 (1.7‐4.7)	2.6 (1.6‐3.7)	5.5 (3.5‐13.2)	.156
Total bilirubin, μmol/L	11.5 (8.6‐14.2)	11.6 (8.9‐14.4)	9.9 (8‐13.6)	.054
Direct bilirubin, μmol/L	3.6 (2.6‐4.7)	3.6 (2.7‐4.4)	3.6 (2.4‐5.2)	.892
Total protein, g/L	68.5 (64‐73.6)	69.6 (64.7‐74)	67.3 (63.3‐69.8)	.029
Globulin, g/L	29.4 (26.1‐33.6)	28.9 (26.3‐33.4)	31.1 (23.8‐33.7)	.708
Albumin, g/L	39 (35.7‐43.1)	39.5 (36.9‐43.6)	36 (31.8‐41.5)	<.001
<34 (n)	30	15	15	<.001
Prealbumin, mg/L	188.4 (133.6‐261.5)	205.5 (143.9‐268)	133.3 (74.1‐181.4)	<.001
<150 (n)	76	47	29	<.001
Alkaline phosphatase, U/L	56 (43‐70)	54.5 (42‐70)	57 (44‐73)	.328
γ‐glutamyl transpeptidase, U/L	24 (15‐39.1)	22.1 (15‐38)	32 (17‐56)	.083
>40 (n)	47	32	15	.044
Alanine aminotransferase, U/L	20 (13‐36.9)	18.8 (12.3‐33)	27 (15‐45.3)	.057
Aspartate aminotransferase, U/L	25 (21‐35)	24 (20‐31)	32 (25‐38)	.085
Blood urea nitrogen, mmol/L	3.7 (3.1‐4.6)	3.6 (3‐4.5)	4.6 (3.4‐5.7)	.641
Creatinine, μmol/L	61.3 (53.4‐72)	61 (53.9‐71.1)	64 (53‐73)	.376
Uric acid, μmol/L	250 (195.5‐330.5)	254.5 (206.8‐336)	217.9 (158‐289.2)	.005
Creatine kinase, U/L	63 (45.3‐95.8)	63 (47‐94.3)	60 (33‐104.5)	.602
Lactate dehydrogenase, U/L	185.5 (156‐232.5)	173.5 (152.8‐220.5)	231.5 (195.5‐362.8)	<.001
>245 (n)	50	27	23	<.001
Potassium, mmol/L	3.9 (3.7‐4.2)	3.9 (3.7‐4.2)	3.8 (3.4‐4.1)	.204
Sodium, mmol/L	140 (138‐141)	140 (139‐142)	137.3 (134.8‐139.7)	.001
Chlorine, mmol/L	105 (103‐107)	105.5 (104‐107.4)	103 (101‐105)	.001
Total calcium, mmol/L	2.1 (2‐2.2)	2.2 (2.1‐2.3)	2.1 (1.9‐2.1)	.001

**FIGURE 1:**
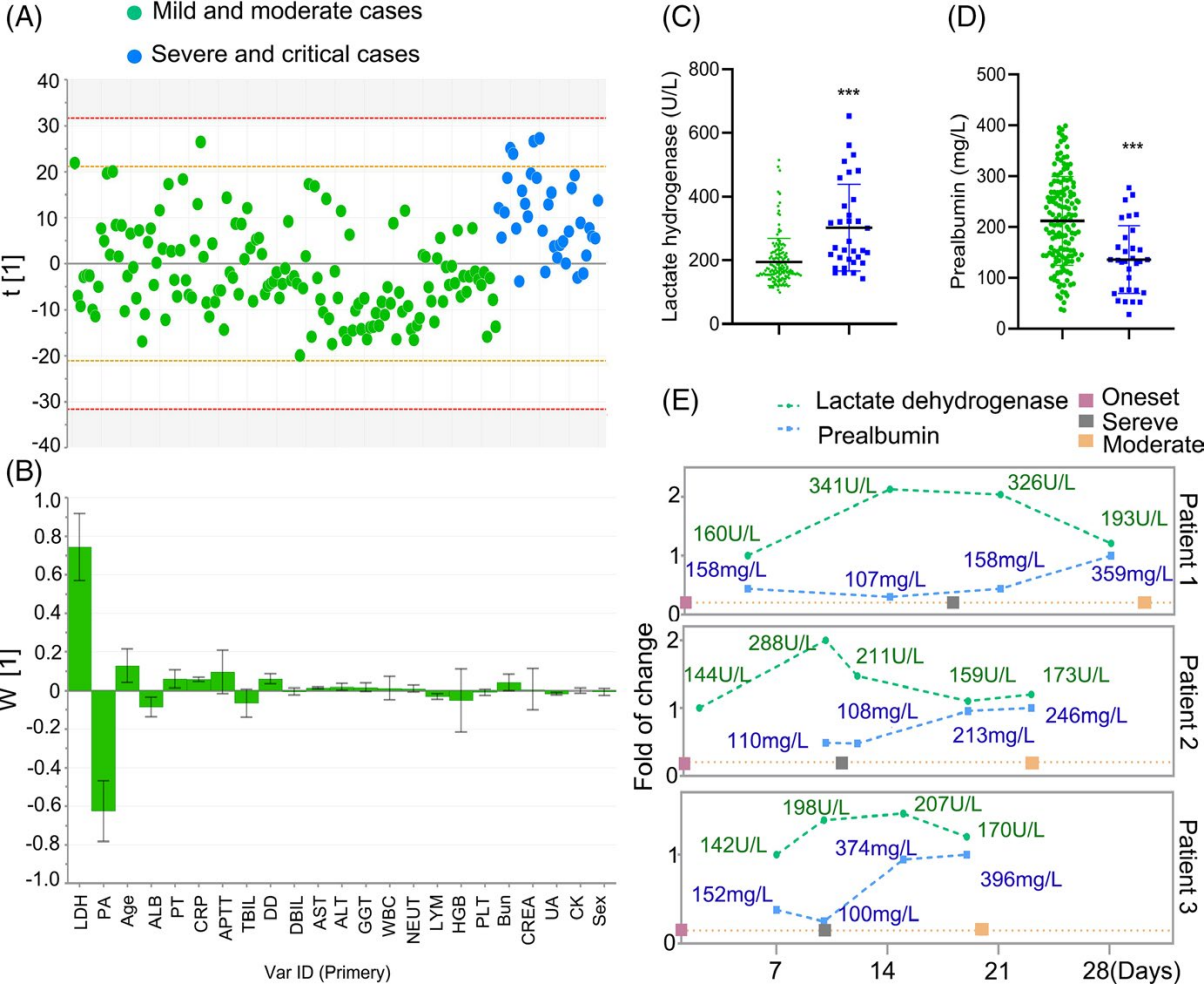
Separation of severe/critical from mild/moderate cases in adult patients with no comorbidities. The scores scatter plot for partial least squares regression discriminant analysis to separate severe/critical from mild/moderate cases (A). PLS‐DA loadings for components ascertained influencing biomarkers (B). The severe/critical cases had a higher LDH (C) and prealbumin (D) levels at admission compared with mild/moderate cases. Dynamic curves of LDH and prealbumin in individual patients with major events during the progression (E). LDH, lactate dehydrogenase; PA, prealbumin; AST, aspartate aminotransferase; APTT, activated partial thromboplastin time; ALB, albumin; PT, prothrombin time; CRP, c‐reactive protein; DD, d‐dimer; TBIL, total bilirubin; DBIL, direct bilirubin; ALT, alanine aminotransferase; GGT, γ‐glutamyl transpeptidase; WBC, white blood cell count; NEUT, neutrophils; LYM, lymphocytes; HGB, hemoglobin; PLT, platelets; Bun: blood urea nitrogen; CREA, creatinine; UA, uric acid; CK, creatine kinase

The data from the patients with no comorbidities were plotted individually in Figure 1. All data points except very few observations fall within the 0.95 Hotelling ellipse (Figure 1A). The strong outliers (8 cases) were distinguished and excluded from the final model. The PLS‐DA model to distinguish the severe/critical from moderate cases was fitted yielding cumulative R2Y (fraction of y‐variation modeled in the component) and Q2 (overall cross‐validated R2Y for the component) values to be equal to 0.22 and 0.2, respectively. The corresponding loading plot for PLS‐DA showed the separation of severe/critical from mild/moderate cases. In terms of contribution to distinguishing severe/critical from mild/moderate cases, the loadings for PC1 illustrated that the significance rank was LDH > prealbumin > age > albumin > prothrombin time > CRP (Figure 1B). LDH and prealbumin were the largest positive and negative loadings responsible for the separation of severe/critical from mild/moderate cases. No differences in the variables including activated partial thromboplastin time (APTT), total bilirubin (TBIL), direct bilirubin (DBIL), γ‐glutamyl transpeptidase (GGT), white blood cell count, neutrophils, hemoglobin, platelets (PLT), creatinine, creatine kinase (CK), and sex were observed between these two groups.

Compared with mild/moderate cases, the levels of LDH were 1.5‐fold (*P* < .001) higher in severe/critical cases; additionally, severe/critical cases had lower levels of prealbumin (*P* < .001) (Figure 1C,D). Among the cases, 3 patients had a progressive disease during the hospitalization. They were classified as a moderate case at admission and progressed to severe disease. After the treatment, they were converted from severe to moderate. Dynamic curves of LDH and prealbumin in these inpatients indicated that the increase or decrease in LDH and prealbumin levels preceded the deterioration or alleviation of respiratory indexes (Figure 1E). Although LDH levels in these patients were within the reference range at admission, the levels were increased before they progressed from moderate to severe cases.

Since the older age has been reported as a risk factor of COVID‐19 severity and mortality,^11^, ^12^, ^13^ we chose age and the top 5 variables extracted from PCA/PLS‐DA analysis to perform multivariable logistic regression analysis (Table 3). The data showed that an increased risk of disease severity was markedly associated with elevated LDH and reduced prealbumin. However, no significant relationship was observed between disease severity and age, albumin, prothrombin time, and CRP in patients with no comorbidities.

Laboratory markers at admission associated with disease severity in COVID‐19 patients with comorbidities. The median age in patients with comorbidities was 61 years (IQR, 50 to 69), and 69% of the patients had fever at the time point of first admission to hospital (Table 1). A total of 28% patients were categorized as severe/critical cases. The most common pattern on chest CT was bilateral patchy shadowing in the adult inpatients (63.9%). Among these cases, 239 (37.6%) patients had hypertension, 145 (22.8%) patients had diabetes, and 90 (14.2%) patients had liver diseases. The treatment and outcome for patients with comorbidities were shown in Table S2. Among the adult cases, 62% of patients received oxygen therapy, and mechanical ventilation was administered in 6.6% (Table 2).

**Table 2:** Clinical characteristics for adult inpatients with comorbidities. Values are medians (interquartile ranges) or numbers (%)

	Adults			
	Total (n = 635)	Mild and moderate (n = 457)	Severe and critical (n = 178)	*P* value
Epidemiological data				
Age, y	61 (50‐69)	58 (47‐67)	66 (60‐72)	<.001
Female	318 (50%)	246 (53.8%)	72 (40.4%)	<.001
Source of infection				
Family source	37 (5.8%)	26 (5.7%)	11 (6.2%)	.813
Community source	7 (1.1%)	3 (0.6%)	4 (2.2%)	.085
Nosocomial infection	5 (0.8%)	5 (1.1%)	0	.161
Others	586 (92.3%)	423 (92.6%)	163 (91.6%)	.675
Hospitalization time, d	17 (10‐25)	16 (10‐24)	18 (8‐27)	.231
Onset time, d	30 (22‐37)	30 (23‐37)	28 (19‐38)	.256
Hypertension	239 (37.6%)	145 (31.7%)	94 (52.8%)	<.001
Diabetes	145 (22.8%)	103 (22.5%)	42 (23.6%)	.707
Respiratory failure	33 (5.2%)	0	33 (18.5%)	<.001
Coronary heart disease	52 (8.2%)	29 (6.3%)	23 (12.9%)	.007
Heart failure	18 (2.8%)	5 (1.1%)	13 (7.3%)	<.001
Liver disease	90 (14.2%)	58 (12.7%)	32 (18%)	.086
Renal failure	20 (3.1%)	10 (2.2%)	10 (5.6%)	.026
Tumor	14 (2.2%)	10 (2.2%)	4 (2.2%)	.964
Cerebrovascular disease	37 (5.8%)	18 (3.9%)	19 (10.7%)	.001
Autoimmune disease	14 (2.2%)	9 (2%)	5 (2.8%)	.518
Prodromal symptoms and signs				
Fever	438 (69%)	303 (66.3%)	135 (75.8%)	.02
Dry cough	380 (59.8%)	270 (59.1%)	110 (61.8%)	.53
Expectoration	59 (9.3%)	32 (7%)	27 (15.2%)	.001
Shortness of breath	148 (23.3%)	90 (19.7%)	58 (32.6%)	.001
Diarrhea	47 (7.4%)	30 (6.6%)	17 (9.6%)	.197
Laboratory tests				
Abnormalities on chest CT				
Ground‐glass opacity	116 (18.3%)	85 (18.6%)	31 (17.4%)	.729
Local patchy shadowing	36 (5.7%)	31 (6.8%)	5 (2.8%)	.052
Bilateral patchy shadowing	406 (6.4%)	286 (62.6%)	120 (67.4%)	.255
Interstitial abnormalities	19 (3%)	14 (3.1%)	5 (2.8%)	.866
White blood cell count, 10^9^/L	5.7 (4.4‐7.3)	5.5 (4.3‐6.9)	6.4 (4.7‐9.1)	<.001
Neutrophils, 10^9^/L	3.9 (2.7‐5.4)	3.5 (2.6‐4.9)	4.9 (3.4‐7.6)	<.001
Lymphocytes, 10^9^/L	1.1 (0.7‐1.5)	1.2 (0.8‐1.6)	0.8 (0.6‐1.3)	<.001
<0.8 (n)	183	100	83	<.001
Eosinophils, 10^9^/L	0.03 (0‐0.1)	0.05 (0.01‐0.1)	0.01 (0‐0.06)	.018
Basophils, 10^9^/L	0.02 (0.01‐0.03)	0.02 (0.01‐0.03)	0.02 (0.01‐0.03)	.36
Red blood cells, 10^12^/L	4.2 (3.9‐4.6)	4.2 (3.9‐4.5)	4.2 (3.9‐4.6)	.854
Hemoglobin, g/L	126 (116‐137)	127 (116.4‐138)	125 (114‐136)	.143
Platelets, 10^9^/L	199 (153‐253)	206.5 (160.5‐258.8)	188.5 (131.8‐243.3)	.038
C‐reactive protein, mg/L	16.8 (3‐68.1)	9.1 (1.8‐36.5)	68.2 (24.5‐130.8)	<.001
>5 (n)	422	263	159	<.001
Prothrombin time, s	13.2 (11.1‐15.2)	12.9 (10.8‐14.8)	14.2 (12.5‐17)	<.001
International normalized ratio	1 (0.9‐1.2)	1.0 (0.9‐1.2)	1.1 (1.0‐1.4)	<.001
Activated partial thromboplastin time, s	35.9 (27.9‐40.4)	35.2 (27.4‐39.8)	37.3 (30.4‐41.8)	.007
Fibrinogen, g/L	3.9 (2.9‐5.1)	3.7 (2.8‐4.7)	4.8 (3.4‐5.6)	<.001
>4 (n)	242	150	92	<.001
D‐dimer, μg/mL	0.6 (0.3‐1.5)	0.5 (0.3‐1.1)	1.4 (0.6‐3.1)	<.001
>0.5 (n)	379	236	143	<.001
Thrombin time, s	17.6 (16.6‐18.5)	17.4 (16.6‐18.3)	17.9 (16.6‐19.1)	.138
Fibrinogen degradation products, μg/mL	3.7 (2.3‐6.3)	3.2 (2.1‐5.1)	6 (4‐15.4)	<.001
Total bilirubin, μmol/L	11.1 (8.1‐15)	11 (8.1‐14.4)	11.4 (8.3‐17.5)	.105
Direct bilirubin, μmol/L	3.7 (2.6‐5.1)	3.5 (2.5‐4.7)	4.5 (3.1‐6.7)	.028
Total protein, g/L	68 (64‐73.5)	68.5 (64.2‐74.1)	67.2 (62.6‐71.9)	.004
Globulin, g/L	31.4 (28‐34.9)	31.3 (27.5‐34.4)	32.6 (28.7‐36.9)	.003
Albumin, g/L	36.8 (33‐40.8)	37.9 (34.2‐41.6)	34.6 (30.8‐37.6)	<.001
<34 (n)	174	102	72	<.001
Prealbumin, mg/L	150 (97.6‐242)	184 (117.1‐260.5)	102 (63‐146.5)	<.001
<150 (n)	277	162	115	<.001
Alkaline phosphatase, U/L	58 (45‐75)	57.5 (44‐75)	58 (46‐76.3)	.28
γ‐glutamyl transpeptidase, U/L	27 (17‐46.2)	24 (16.7‐44)	33.5 (20.2‐56)	.007
>40 (n)	180	119	61	.039
Alanine aminotransferase, U/L	23 (14.4‐37.1)	21 (13.9‐34.2)	27 (16‐43)	.024
Aspartate aminotransferase, U/L	29.6 (22‐41.2)	27.4 (20‐38)	38 (27‐51.8)	<.001
>40 (n)	163	96	67	<.001
Blood urea nitrogen, mmol/L	4.3 (3.4‐5.9)	4.1 (3.3‐5.4)	5.4 (3.8‐7.9)	<.001
Creatinine, μmol/L	64 (53.0‐82)	62 (52.6‐77.7)	72 (57‐96)	.147
Uric acid, μmol/L	270 (206.8‐345)	277 (208.9‐345)	246.3 (193‐343.8)	.654
Creatine kinase, U/L	64 (43‐113.5)	60 (41.5‐95.5)	87.5 (46.5‐184.3)	.008
>200 (n)	67	31	36	<.001
Lactate dehydrogenase, U/L	217 (170.5‐298.3)	197.5 (161‐249)	335.5 (235.5‐453.5)	<.001
>245 (n)	237	116	121	<.001
Potassium, mmol/L	3.9 (3.6‐4.2)	3.9 (3.6‐4.3)	3.7 (3.5‐4.1)	.066
Sodium, mmol/L	140 (137‐141.6)	140 (138‐142)	138.6 (135.8‐141.1)	.004
Chlorine, mmol/L	105 (102‐107)	105 (102.3‐107.5)	103.2 (101‐107)	.013
Total calcium, mmol/L	2.1 (2.0‐2.2)	2.1 (2.0‐2.2)	2 (1.9‐2.1)	<.001

**FIGURE 2:**
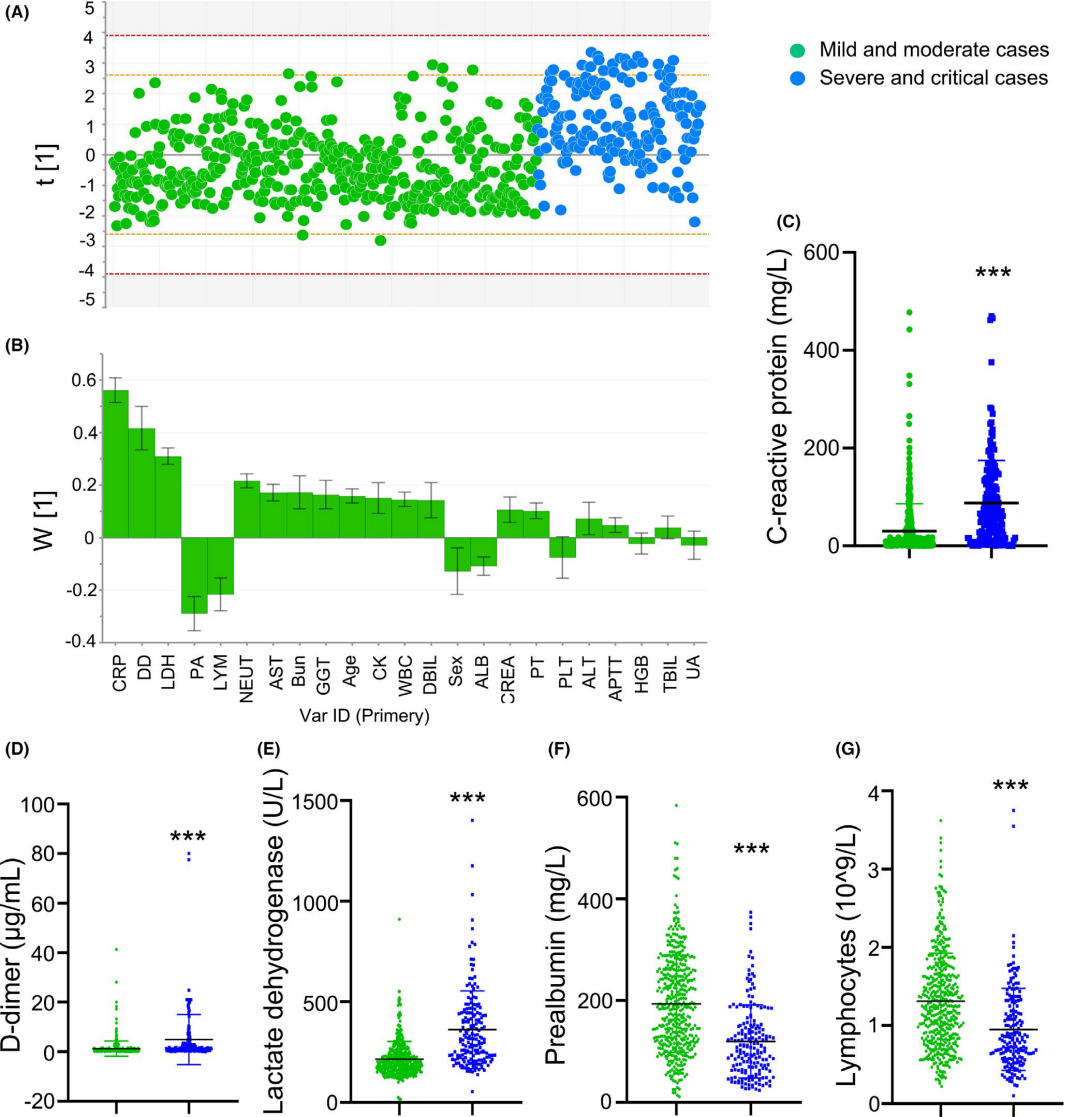
Separation of severe/critical from mild/moderate cases in total adult patients. The scores scatter plot for partial least squares regression discriminant analysis to separate severe/critical from mild/moderate cases (A). PLS‐DA loadings for components ascertained influencing biomarkers (B). C‐reactive protein (C), D‐dimer (D), lactate dehydrogenase (E), prealbumin (F), and lymphocytes (G) levels were higher in severe/critical cases compared with mild/moderate cases. LDH, lactate dehydrogenase; PA, prealbumin; AST, aspartate aminotransferase; APTT, activated partial thromboplastin time; ALB, albumin; PT, prothrombin time; CRP, c‐reactive protein; DD, d‐dimer; TBIL, total bilirubin; DBIL, direct bilirubin; ALT, alanine aminotransferase; GGT, γ‐glutamyl transpeptidase; WBC, white blood cell count; NEUT, neutrophils; LYM, lymphocytes; HGB, hemoglobin; PLT, platelets; Bun: blood urea nitrogen; CREA, creatinine; UA, uric acid; CK, creatine kinase; OR, Odds ratio; CI, Confidence interval; Ref, reference

The data were plotted individually in Figure 2. The observations out of the 0.95 Hotelling ellipse (1 cases) were distinguished and excluded from the final model (Figure 2A). The PLS‐DA model to distinguish the severe/critical from mild/moderate cases was fitted generating cumulative R2Y (0.25) and Q2 (0.24). In terms of contribution to the separation of severe/critical from mild/moderate cases, the loadings for PC1 illustrated that the significance rank was CRP > D‐dimer > LDH > prealbumin > lymphopenia (Figure 2B). CRP was the largest positive loading responsible for the separation of observations, while prealbumin was the largest negative loading responsible for the separation.

Compared with mild/moderate cases, the levels of CRP, D‐dimer, and LDH were significantly higher in severe/critical cases; additionally, severe/critical cases had reduced prealbumin and lymphocytes levels (Figure 2C‐g).

We chose age and the top 5 variables extracted from PCA/PLS‐DA analysis including CRP, D‐dimer, LDH, prealbumin, lymphopenia for multivariable logistic regression model. The data showed that the increased odds of disease severity were associated with age, elevated LDH, and reduced prealbumin in patients with comorbidities; however, CRP, D‐dimer, and lymphocytes were not associated with disease severity (Table 3).

**Table 3:** Multivariate logistic analysis of risk factors for patient

	Odds ratio	95%, CI	*P* value
Risk factors for patients without comorbidities on severity			
Lactate dehydrogenase (per 1 U/L increase)	1.008	1.003‐1.013	.001
Prealbumin (per 1 mg/L increase)	0.991	0.985‐0.998	.010
Age (per year increase)	1.028	0.992‐1.065	.125
C‐reactive protein (per 1 mg/L increase)	1.021	0.938‐1.110	.634
Albumin (per 1 mg/L increase)	0.993	0.980‐1.006	.300
Prothrombin time (per second increase)	1.113	0.912‐1.358	.293
Risk factors for patients with comorbidities on severity			
Age (per year increase)	1.028	1.011‐1.045	.001
Lactate dehydrogenase (per 1 U/L increase)	1.006	1.004‐1.008	<.001
Prealbumin (per 1 mg/L increase)	0.997	0.994‐1.000	.038
C‐reactive protein (per 1 mg/L increase)	1.002	0.999‐1.006	.157
D‐dimer (per 1 μg/mL increase)	1.021	0.990‐1.053	.195
Lymphocytes (per 10^9^/L increase)	0.898	0.583‐1.383	.625

Laboratory markers at admission associated with mortality risk in COVID‐19 patients with comorbidities.

The proportion of death was 8.98% (n = 57) in patients with comorbidities, while only 5 of deaths were identified in patients with no comorbidity. Thus, we performed multivariable Cox proportional hazards regression analysis to evaluate the association of laboratory makers at admission with short‐term mortality risk in patients with comorbidities. The data from Cox proportional hazards regression analysis revealed that the mortality risk was associated with age, LDH, CRP, D‐dimer, and lymphopenia in cases with comorbidities (Table 4). No significant correlation was observed between prealbumin levels and mortality risk.

**Table 4:** Multivariate Cox hazard analysis of risk factors for patient

	Hazard ratio	95%, CI	*P* value
Risk factors for patients with comorbidities on death			
Age (per year increase)	1.038	1.008‐1.069	.012
Lactate dehydrogenase (per 1 U/L increase)	1.003	1.002‐1.004	.001
C‐reactive protein (per 1 mg/L increase)	1.001	1.000‐1.002	.021
D‐dimer (per 1 μg/mL increase)	1.020	1.003‐1.038	.020
Lymphocytes (per 10^9^/L increase)	0.229	0.101‐0.520	.001
Prealbumin (per 1 mg/L increase)	0.997	0.992‐1.002	.209

**FIGURE 3:**
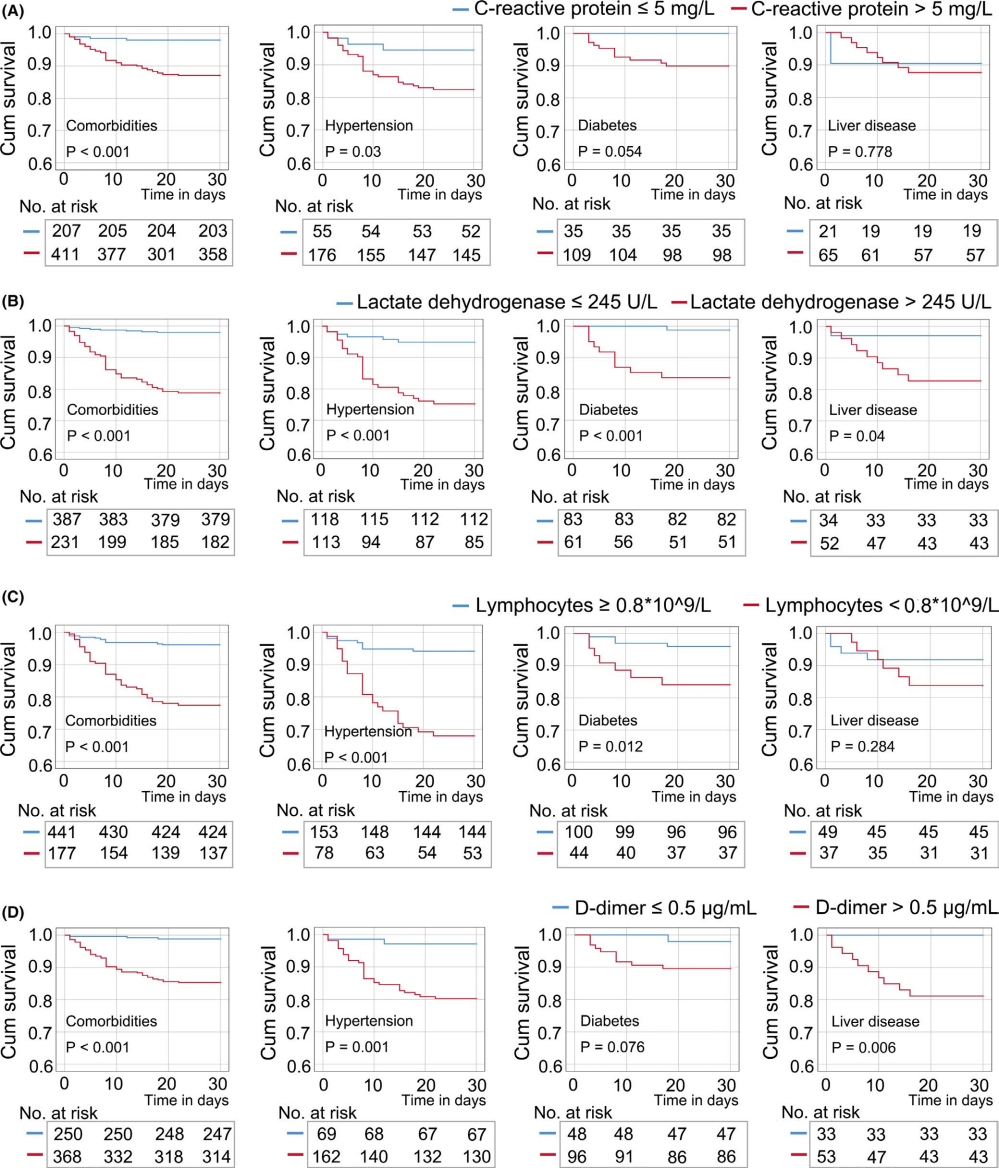
Kaplan‐Meier survival analysis in patients with comorbidities according to levels of C‐reactive protein (A), lactate dehydrogenase (B), lymphocytes (C), and D‐dimer (D). A, In the total cases with comorbidities or in the subgroup of hypertension, patients with high C‐reactive protein (CRP) levels were at higher mortality risk than those patients with normal levels of CRP. B, In the subgroups of hypertension, diabetes, and liver diseases, patients with high lactate dehydrogenase (LDH) levels were at higher mortality risk than those patients with normal levels of LDH. C, In the subgroups of hypertension and diabetes, patients with reduced lymphocytes were at higher mortality risk than those patients with normal levels of lymphocytes. D, In the subgroups of hypertension and liver diseases, patients with high D‐dimer levels were at higher mortality risk than those patients with normal levels of D‐dimer

Among patients with comorbidities, the Kaplan‐Meier survival curve showed a lower survival rate in patients with elevated CRP (>5 mg/L), LDH (245 U/L), and D‐dimer (>0.5 μg/mL); additionally, patients with reduced lymphocytes (<0.8 × 10^9^/L) also had a lower survival rate (Figure 3A‐D). In the subgroup of hypertension, the survival rate was significantly lower in patients with increased CRP, LDH, and D‐dimer, or decreased lymphocytes. In the subgroup of diabetes, patients with elevated LDH or reduced lymphocytes had a lower survival rate compared with the controls; meanwhile, a trend toward lower survival rate was observed in patients with elevated CRP and D‐dimer. In the subgroup of liver diseases, patients with elevated LDH and D‐dimer had a lower survival rate compared with the controls; however, no significant change in survival rate was observed in patients with elevated CRP or reduced lymphocytes.

## 4 DISCUSSION

This study demonstrated the laboratory markers at admission associated with disease severity and mortality of COVID‐19 patients. The predictors for the mortality of COVID‐19 were different among the patients with or without comorbidities. We showed that LDH was a reliable predictor associated with the severity and mortality of COVID‐19 patients with different medical conditions. The inflammation marker CRP was a risk factor associated with short‐term mortality in patients with hypertension, but not liver diseases. The coagulation system marker D‐dimer was an independent risk factor for death in patients with liver diseases. These results suggest that there are multiple pathophysiological mechanisms following COVID‐19 infection, and that personalized treatment (eg, anti‐inflammatory and thromboembolic prophylaxis) based on individual medical condition is required to reduce the mortality risk.

The PCA/PLS‐DA models generated from patients with or without comorbidities indicated that both LDH and prealbumin were important indicators responsible for the separation of severe/critical cases from mild/moderate cases. On the basis of subgroup analysis, LDH was a reliable and dominant predictive factor associated with poor prognosis in patients with different medical conditions (eg, hypertension, diabetes, liver diseases). The elevated LDH was found in COVID‐19 patients in China and Iran ^14^, ^15^ and has been reported to be associated with disease severity.^16^, ^17^, ^18^ In addition, a low prealbumin level in the patients with COVID‐19 infection was associated with poor prognosis in both China and North American patients.^19^ The low level of prealbumin in COVID‐19 severe cases suggests that nutritional support may be important for supportive therapy during COVID‐19 infection.

The previous studies showed that CRP and D‐dimer were associated with disease severity or death.^20^, ^21^, ^22^, ^23^, ^24^ However, our findings from PCA/PLS‐DA analysis were inconsistent in patients with or without comorbidities. The hyperinflammation was an important contributor in distinguishing between severe/critical and mild/moderate cases in patients with comorbidities. The data from Cox regression analysis revealed that CRP was an independent risk factor associated with death in cases with comorbidities. These results indicated that the comorbidities might aggravate the inflammation response to COVID‐19 infection, leading to the death. Based on survival analysis, the anti‐inflammatory treatment may be required in hypertension patients with high levels of CRP to reduce the death risk.

The risk factors for COVID‐19 mortality are not identical in patients with different comorbidities. The previous studies showed that elevation of D‐dimer was associated with disease severity and mortality in COVID‐19 patients due to the increased risk for the development of pulmonary embolism.^20^, ^25^, ^26^, ^27^, ^28^ The data that elevated D‐dimer levels were accompanied with a parallel rise in markers of inflammation (eg, CRP) ^29^ supported the hypothesis that a severe inflammatory response subsequently activated coagulation and thrombin generation. Notably, the survival analysis based on our dataset showed that the reduced survival rate in patients with liver diseases was associated with the increased D‐dimer, but not the elevated CRP. The thrombotic microangiopathy and microcirculatory impairment due to the endothelial cell injury induced by COVID‐19 could be aggravated by liver dysfunction as most coagulation factors, anticoagulants, and fibrinolytic proteins are synthesized in the liver. The coagulation disorders could be an important mechanism in COVID‐19 patients with liver disease rather than cytokine storm.

This study has a number of limitations. For the evaluation of disease severity, we did not have information of qPCR Ct value to assess the viral loads. The relationship between the indicators and viral loads needs to be elucidated in future studies to gain a better understanding.

In conclusion, LDH and prealbumin were relatively reliable markers for the assessment of disease severity in adult patients regardless of the comorbidities. CRP was an independent risk factor associated with short‐term mortality in patients with hypertension, but not liver diseases. The laboratory biomarkers for disease prognosis of COVID‐19 in patients with different comorbidities need to be further refined. Although the laboratory indicators are non‐specific, the features of the biomarkers could be helpful to understand the pathological mechanism of COVID‐19 infection and for therapy management.

## AUTHORS' CONTRIBUTIONS

ZC, FZ, JM, and JY designed research; ZC, FZ, WH, ZZ, BL, QC, CL, LW, JM, and JY conducted research; ZC, FZ, QY, JM, and JY analyzed data; FZ and JY wrote the paper. JM and JY had primary responsibility for final content. All authors read and approved the final manuscript.

## ETHICAL APPROVAL

This was a retrospective case series study, and no patients were directly involved in the study design or in setting the research questions or the outcome measures directly. The study was in accordance with the ethical standards of the institutional and national research committee and with the 1964 Helsinki Declaration and its later amendments or comparable ethical standards. The local ethics committee of the People's Hospital of Jiayu County approved the study (reference 2020‐02).

## Supporting information

Tab S1‐S2
