# Supplementary material for: Laboratory markers associated with COVID‐19 progression in patients with or without comorbidity: A retrospective study
Source: J Clin Lab Anal. 2020 Oct 28;35(1):e23644. doi: 10.1002/jcla.23644 (PMC7645968; doi:10.1002/jcla.23644)
Supplement: Supplementary file 1 — Tab S1‐S2 [file JCLA-35-e23644-s001.docx]

Table S1 Treatment and outcome of adult inpatients without comorbidities

|  | Total  (n = 201) | Mild and Moderate  (n = 158) | Severe and critical  (n = 43) |
| --- | --- | --- | --- |
| Treatment |  |  |  |
| Antiviral treatment |  |  |  |
| Interferon alpha inhalation | 44 | 29 | 15 |
| Lopinavir/ritonavir | 44 | 33 | 11 |
| Hydroxychloroquine | 11 | 9 | 2 |
| Arbidol | 144 | 119 | 25 |
| Antibiotics | 171 | 131 | 40 |
| Corticosteroid | 56 | 24 | 32 |
| Gamma globulin | 48 | 32 | 16 |
| Traditional Chinese medicine | 172 | 134 | 38 |
| Oxygen therapy | 116 | 73 | 43 |
| Mechanical ventilation | 6 | 0 | 6 |
| Prognosis |  |  |  |
| Discharge | 196 | 158 | 38 |
| Death | 5 | 0 | 5 |

Table S2 Treatment and outcome of adult inpatients with comorbidities

|  |  | Adults |  |
| --- | --- | --- | --- |
|  | **Total**  **(n = 635)** | **Mild and moderate**  **(n = 457)** | **Severe and critical**  **(n = 178)** |
| Treatment |  |  |  |
| Antiviral treatment |  |  |  |
| Interferon alpha inhalation | 160 | 102 | 58 |
| Lopinavir/ritonavir | 99 | 74 | 25 |
| Hydroxychloroquine | 11 | 8 | 3 |
| Arbidol | 424 | 310 | 114 |
| Antibiotics | 537 | 374 | 163 |
| Corticosteroid | 236 | 136 | 100 |
| Gamma globulin | 186 | 100 | 86 |
| Traditional Chinese medicine | 514 | 386 | 128 |
| Oxygen therapy | 394 | 248 | 146 |
| Mechanical ventilation | 42 | 6 | 36 |
| Prognosis |  |  |  |
| Discharge | 578 | 455 | 123 |
| Death | 57 | 2 | 55 |
